# Exploring the Cell Biological and Functional Effects of the First Disease Associated KCC1 Genetic Variant

**DOI:** 10.1002/jcp.70124

**Published:** 2025-12-19

**Authors:** Meye Bloothooft, Jiahui Huang, Mira Hamze, Marien J. C. Houtman, Teun P. de Boer, Peter M. van Hasselt, Gerhard F. Ecker, Christophe Porcher, Igor Medina, Marcel A. G. van der Heyden

**Affiliations:** ^1^ Department of Medical Physiology, Division of Heart & Lungs University Medical Center Utrecht Utrecht the Netherlands; ^2^ Department of Pharmaceutical Sciences University of Vienna Vienna Austria; ^3^ INMED, INSERM Aix‐Marseille Université Marseille France; ^4^ Department of Metabolic Diseases, Wilhelmina Children's Hospital Utrecht University Medical Center Utrecht Utrecht the Netherlands

**Keywords:** disease associated, genetic variant, KCC1, potassium chloride cotransporter, SLCA12A4

## Abstract

The potassium chloride cotransporter 1 (KCC1) is ubiquitously expressed and essential for regulating cellular fluid balance. We identified a patient carrying a genetic variant (E1065K) in the KCC1 coding gene SLC12A4. This study explored the impact of the variant in ectopic cell systems and enhanced the understanding of cell biological properties of the KCC1 protein. KCC1 WT and E1065K DNA expression constructs were transfected in HEK293T, EPI7 or COS7 cells. KCC1 protein expression levels, glycosylation, intracellular trafficking, half‐life and protein localization were determined with western blot and immunofluorescence microscopy. Molecular docking investigated interactions within the cotransporter. Cotransporter activity was tested with NH_4_^+^ flux measurements. The variant reduces interactions within the cotransporter and functional activation decreases in hypotonic conditions. Other cell biology characteristics with respect to protein expression level, half‐life or subcellular localization did not show any statistical difference between KCC1 WT and E1065K. However, this data provided new characteristics of KCC1 protein. Altogether, these findings are the first description of a potential pathogenic human variant in the KCC1 protein.

## 1 Introduction

The K^+^‐Cl^−^ cotransporter 1 (KCC1/SLC12A4), mediates coupled efflux of potassium and chloride ions across the cell plasma membrane. This cotransporter is ubiquitously expressed and important for chloride and potassium homeostasis and fluid balance in cells. When a cell is under stress, like hypotonic conditions, the cell will swell, and subsequently KCC1 becomes activated, thereby restoring cell shape (Gillen et al. 1996; Lauf et al. 1992; Mercado et al. 2000; Gillen and Forbush 1999). Thus far, the physiological function of KCC1 is mainly described for red blood cells, but KCC1 is expressed in many other tissues and organs, e.g., colon (Sangan et al. 2000), bone (Kajiya et al. 2006), and kidney (Liapis et al. 1998). A cotransporter with such an important function for cell integrity maintenance is expected to be linked to pathologies, like sickle cell disease. However, for a long time, KCC1 was regarded as a housekeeping gene, without any link to human pathology (Gillen et al. 1996; Garneau et al. 2019).

KCC1 is part of the chloride cation cotransporter (CCC/SLC12) family. The KCC family comprises of four different members, KCC1‐4. KCC1s amino acid sequence identity is respectively 78% and 67% identical to KCC3 and KCC2. KCC1 consists of 12 transmembrane domains with a 120 amino acid‐long loop between domains 5 and 6, where 2 glycosylation sites, Asn312 and Asn361, are positioned. The extracellular loop with glycosylation sites distinguishes the KCC proteins from other members of the cation chloride cotransporters (CCC) family, in which it is much shorter (Gillen et al. 1996; Liu et al. 2019; Payne et al. 1996). As functional cotransporters, KCC1 proteins adopt a dimeric assembly with the C‐ and N‐terminus residing intracellularly (Liu et al. 2019). Although these structural properties of KCC1 are quite extensively described, its cell‐biological properties are not.

Over recent years, research has focused on unraveling the (patho)physiology of KCC1. Humanized mouse models carrying a KCC1 mutation showed enhanced progression of sickle cell disease (Brown et al. 2015). It was also found that cotransport activity of KCC1 in *Xenopus* oocytes requires the presence of the C‐terminus and N‐terminus (Casula et al. 2001). For two other members of the KCC family, pathogenic variants were discovered; KCC2 mutations cause epilepsy (Fukuda and Watanabe 2019) and KCC3 mutations lead to Andermann syndrome (Uyanik et al. 2006). Combining the important physiological role of KCC1 and mutations observed in other KCC family proteins, might imply that KCC1 could also associate to human disease. However, such linkage is not described yet, as reported in a 2019 review (Garneau et al. 2019).

Our recent discovery of a novel human variant of unknown significance in the SLC12A4 gene, which encodes for the KCC1 protein, might shed light on the possible link of KCC1 to human disease. The patient showed symptoms reminiscent of intestinal dysmotility, extrapyramidal movement disorder and headaches. The variant, KCC1 E1065K, replaces a negatively charged glutamic acid for a positively charged lysine in the cytoplasmic C‐terminus of the protein. Previous research showed that the truncation of the KCC1 C‐terminus induces loss‐of‐function. Truncation of only 8 amino acids at the end of the amino acid sequence showed no decrease in surface expression, but showed only low‐level activity under isotonic conditions and abolished stimulation at hypotonic conditions (Casula et al. 2001; Lauf et al. 2001). The C‐terminus has many important functional domains that, for example, are responsible for transport activity of the transmembrane domain (Chew et al. 2021; Rinehart et al. 2009). Therefore, an amino acid change in the C‐terminus might affect KCC1 protein function.

In this study, the (sub)cellular expression and functionality of KCC1 wild‐type (WT) and E1065K were investigated. We discovered an explanation for how KCC1 E1065K might be linked to disease and simultaneously aimed to increase the knowledge on cell biological properties of the KCC1 protein.

## 2 Materials and Methods

### 2.1 Expression Constructs

The pCMV6‐Entry KCC1‐Myc‐Flag construct (transcript variant 2) was obtained (Origene, Rockville, USA, RC228636) and the E1065K variant (GAG > AAG) was made using the QuikChange Site‐Directed Mutagenesis kit (Agilent, Santa Clara, USA, 200519) according to the manufacturer's instructions using 100 ng template (SE: CTCGAGGTGCTGACCAAGGGCCTTGAGC, AS: GCTCAAGGCCCTTGGTCAGCACCTCGAG). Successful mutagenesis was confirmed using the EZ‐seq sequencing service (Macrogen Europe, Amsterdam, NL).

The non‐tagged KCC1 encoding coding region of human KCC1‐2 transcript variant 2 (KCC1‐2‐WT; NM_001145961.2) and its E1065K variant were obtained by Myc‐Flag removal using restriction enzymes. The non‐tagged KCC1 encoding coding region of human KCC1‐1‐WT transcript variant 1 (NM_005072.5) and its E1065K variant KCC1‐1‐E1065K were synthesized by GenScript.com and subcloned in pcDNA3.1(‐). The sequences of the constructs, as well as the cDNAs, are available on request.

### 2.2 Cell Culture and DNA Transfection

HEK293T and COS7 cells (RRID:CVCL_0063 & CVCL_0224) were cultured in Dulbecco's modified Eagle medium (DMEM, Capricorn Scientific, Ebsdorfergrund, DE, DMEM‐HXA). EPI7 cells (Mummery et al. 1985) were cultured in DMEM:F12 (1:1) medium (Gibco, Green Island, USA, 11320‐033). Both media were supplemented with 10% fetal bovine serum (FBS, Corning, Amsterdam, NL, 35‐015‐CV), 2 mM l‐glutamine (Lonza, Basel, CH, 17‐605E), 50 U/mL penicillin and 50 mg/mL streptomycin (Lonza, Basel, CH, DE17‐602E) and passaged two times a week. KCC1 WT or KCC1 E1065K with Flag tag DNA constructs were transfected into cells using polyethylenimine (Mw 25000) (Polysciences, Warrington, USA) for 24 h prior to protein isolation or treatment. Western blot analyses confirm that both variants become equally expressed in all three used cell lines (Figure S1).

For functional assays, HEK293T cells were cultured on 12 mm glass coverslips in 35 mm dishes, in MEM:DMEM (1:1) supplemented with 8% FBS, 45% glucose (1:200) and 10 IU/ml penicillin/streptomycin. The cells were transfected with 300 µL Opti‐MEM, 8 µL Lipofectamine 2000, 2 µL CombiMag (OZ Biosciences, Marseille, FR) and 2 µg DNAs of interest (Invitrogen, Waltham, USA). After 15 min incubation at room temperature, the mix was added to the culture. Dishes were then placed on a magnetic plate for 2 h at 37°C with 5% CO_2_. Transfection was ended by replacing 50% of the solution with a fresh medium. Cells were used 48‐72 h post‐transfection. Generally, co‐transfection of two plasmids was used per well, pH‐sensor (0.4 µg) and either one of the following constructs: KCC1 WT, KCC1 E1065K, or empty vector as a control (1.6 µg).

### 2.3 Compounds

Tunicamycin from *Streptomyces* sp. (Sigma‐Aldrich, Zwijndrecht, NL, T7765) was used as an inhibitor of N‐linked glycosylation. It was dissolved in DMSO at a concentration of 10 mg/mL, and the final working concentration was 5 µg/mL. Cycloheximide (Sigma‐Aldrich, Zwijndrecht, NL, C7698) was used as an inhibitor of protein synthesis. It was dissolved in sterile filtered water at a concentration of 5 mg/mL and the final concentration was 200 µg/mL. Dynasore (Sigma‐Aldrich, Zwijndrecht, NL, D7693) was used as an inhibitor of dynamin‐dependent endocytosis. It was dissolved at a concentration of 50 mM in DMSO, and the final concentration was 10 µM. Chlorpromazine (Sigma‐Aldrich, Zwijndrecht, NL, C8138) was used as an inhibitor of clathrin‐mediated endocytosis. It was dissolved at a concentration of 10 mM in sterile filtered water, and the final concentration was 10 µM. Chloroquine (Sigma, St. Louis, MO, USA, C6628) was used as a lysosomal inhibitor. It was dissolved at a concentration of 10 mM in sterile filtered water, and the final concentration was 10 µM. All compounds were aliquoted and stored at −22°C. VU0463271 (Euromedex, Souffelweyersheim, France, ref: TA‐T17243), bumetanide (Sigma‐Aldrich, Zwijndrecht, NL, B3023), and amiloride (Sigma‐Aldrich, Zwijndrecht, NL, A7410) used for NH_4_^+^ tests were prepared as 20 mM stocks in DMSO the day of the experiment and dissolved 2000‐fold in recording solutions. Ouabain (Sigma‐Aldrich, Zwijndrecht, NL, O3125) was dissolved directly in the recording solution.

### 2.4 SDS‐PAGE Western Blot

Cells were washed twice with phosphate buffered saline (Lonza, Basel, CH, BE17‐516F) and lysed by adding 200 µL buffer D lysis buffer (20 mM HEPES, 125 mM NaCl, 10% glycerol, 1 mM EDTA, 1 mM EGTA, 1 mM dithiothreitol, 1% Triton X‐100, 10 mM PMSF, 1:100 aprotinin, pH 7.6) per 70–100% confluent 60 mm dish on ice. Protein concentration per sample was determined using the Pierce™ BCA Protein Assay Kit (ThermoFisher Scientific, Waltham, USA, 23225). Protein lysate (30 µg) was mixed with sterile filtered water and 5x Laemmli sample buffer and incubated for 5 min at 37°C. Samples were separated on a 7% SDS‐PAGE gel and transferred on a 0.45 µm nitrocellulose membrane (Bio‐Rad Laboratories, Veenendaal, NL, 162045). Ponceau S staining was used for visualization of equal loading and quantification, which thus is not dependent on a single reference protein. Subsequently, the membranes were blocked by 5% Protifar in Tris‐buffered saline/Tween‐20 (TBST: 20 mM Tris‐HCl, 150 mM NaCl, 0.05% Tween‐20 (v/v)) for 2 h. Membranes were washed 5 times with TBST before incubation with Flag primary antibody (Abcam, Cambridge, UK, ab1162, RRID:AB_298215) 1:1000 o/n followed by 5 additional washes with TBST and subsequent addition of 1:7000 anti Rabbit HRP secondary antibody (Bio‐Rad Laboratories, Veenendaal, NL, 170‐6515, RRID:AB_11125142) for 2 h. Final detection of the KCC1 protein bands was performed using ECL prime detection reagent (Cytiva, Marlborough, USA, RPN2232). Signal detection was done with the ChemiDOCXRS Bio‐Rad Laboratories, Veenendaal, NL) and analysis was performed using Image Lab 6.1 software (Bio‐Rad Laboratories, Veenendaal, NL). Each blot was normalized to the control lane, enabling comparison of multiple blots.

### 2.5 Immunofluorescence Microscopy

COS7 and EPI7 cells were cultured on 0.1% gelatin/ddH_2_O‐coated 15 mm coverslips prior to transfection. Twenty‐4 h after transfection or treatment the coverslips were washed twice with PBS at RT. Cells were fixated with 3% paraformaldehyde/PBS pH 7.4 for 25 min, permeabilized with 0.5% Triton X‐100/PBS for 3 min, and quenched twice with 50 mM glycine/PBS for 10 min. After incubation with NET‐gel (0.25% gelatin, 50 mM Tris, 150 mM NaCl, 5 mM EDTA, 0.05% Igepal, 0.02% NaN_3_) twice for 10 min primary antibody was added. Flag antibody (Abcam, Cambridge, UK, ab1162, RRID:AB_298215, 1:2000 for EPI7 cells, 1:2500 for COS7 cells) for KCC1, anti‐Pan Cadherin (Sigma Aldrich, Zwijndrecht, Netherlands, C‐1821, Mouse, RRID:AB_476826, 1:600 for EPI7 cells, 1:400 for COS7 cells) antibody for plasma membrane were used, for at least 2 h at RT. After 5 times washing with NET‐gel Alexa Fluor 568 1:1000 and Alexa Fluor 488 1:500 (both Invitrogen, Waltham, USA, A‐11011, RRID:AB_143157 and A‐32723, RRID:AB_2633275) were added as secondary antibodies for 2 h. Coverslips were mounted with Vectashield (Vector Laboratories Inc., Burlingame, USA, H1000). Samples were stored at −22°C. Images were made using a Nikon Eclipse 80i light microscope with a 60x oil immersion lens. NIS elements was used as imaging software (Nikon Europe BV., Amsterdam, NL).

### 2.6 NH_4_^+^ Flux Assay

HEK293T cells were transiently transfected with the ratiometric fluorescent probe composed of pH‐independent mCherry and pH‐sensitive pHluorine (called a pH sensor) (Hamze et al. 2024). For recordings, coverslips were placed in a recording chamber and perfused with either an isotonic extracellular HEPES‐buffered solution (HBS) containing (in mM): 150 NaCl, 2.5 KCl, 5 HEPES, 2.0 CaCl_2_, 2.0 MgCl_2_ and 10 glucose, at pH 7.4 or a hypotonic HBS containing (in mM): 75 NaCl, 1.25 KCl, 2.5 HEPES, 1.0 CaCl_2_, 1.0 MgCl_2_ and 5 glucose at pH 7.4. Both solutions were routinely supplemented with bumetanide (10 µM) to block NKCC1, ouabain (100 µM) to inhibit Na⁺/K⁺‐ATPase, and amiloride (10 µM) to inhibit Na⁺/H⁺ and Na⁺/HCO₃⁻‐dependent exchangers. The ratiometric fluorescence of pHluorine/mCherry (F_pH_/F_mCh_) was measured using an epifluorescence imaging setup on an inverted Olympus IX71 microscope (Olympus, Tokyo, JP) with a FITC/CY3 Dualband ET Filterset and additional single‐band filters. pH‐sensitive pHluorine fluorescence (F480) was obtained with a 480/20 excitation filter and 520/40 emission filter, while pH‐insensitive mCherry fluorescence (F577) was obtained with a 577/25 excitation filter and 645/75 emission filter. Fluorescence was sampled at 0.1 Hz using a CoolSNAPHQ CCD camera and MetaMorph software (version 7.7.5.0., Molecular Devices Corp, San Jose, CA, USA). Excitation lasted 100 ms for F480 and 50 ms for F577. Recordings were performed with a 40× objective (NA 0.6). Baseline fluorescence was acquired for 5 min, followed by a 6 min perfusion with 10 mM NH_4_Cl solution and 2–5 min washout imaging. The F_pH_/F_mCh_ ratio was calculated offline from the images, and the acidification rate (ΔR/min) was determined by the change in F_pH_/F_mCh_ values between 0.5 and 5 min during NH_4_^+^ perfusion as described previously (Hamze et al. 2024; Järvelä et al. 2024).

### 2.7 Statistics

No statistical methods predetermined sample sizes. All average values are presented as mean with standard deviation (sd). All statistical tests were performed with Prism 9 (version 9.3.0 (345), GraphPad software, San Diego, USA). Statistics on western blot data were performed with either one‐way ANOVA with Tukey post‐hoc test or two‐way ANOVA with Sidak correction.

For the flux assay, the consistency was assured by repeating trials in different cell cultures from at least three passages per condition. Normal distribution was assessed with the Shapiro–Wilk test. For normally distributed data, we used one‐way ANOVA with the Holm–Sidak post‐hoc test; for non‐normal data, we used the Kruskal–Wallis test with Dunn's post‐hoc test. Statistical analyses were conducted on the data from all cells.

### 2.8 Variant Conformer Modeling

Comparative models were generated using Modeler (Šali and Blundell 1993) to explore potential conformers of the variants. The single template approach was applied with the ‘AutoModel’ function. In the optimization and refinement steps, both standard and enhanced protocols were applied. In the enhanced protocol, the schedule was set as slow, and the iteration was set as 300 to allow a higher degree and duration of the optimization process. The enhanced refinement was controlled with the degree of the MD refinement of the model, which was set as very slow to allow an increased amount of MD annealing in the refinement process.

### 2.9 Electrostatic Map Creation

To create an electrostatic map around the residue of interest, the protein must be prepared to add the missing hydrogen properly. The Protonate 3D application from Molecular Operation Environment (MOE) (Molecular Operating Environment MOE 2024) assigns protonation states by optimizing the titration‐free energy of all titratable groups in an all‐atom model of a macromolecular structure. The Generalized Born/Volume Integral (Labute 2008) electrostatics model was used for detecting longer‐range interactions and solvation effects. The ‘Atom set’ was defined as the whole structure. The ‘Protect group’ was set as None, while the ‘Precise group’ was set as the residues within the range of two‐time selection extension. The selection was conducted by expanding the selection two times around the residue on position 1065. A residue was included as long as the residue was within 4.5Å of the selection. In this way, the Arginine‐rich environment was included in the analysis. The surface was mapped in MOE with the Molecular Surface option by defining the ‘Color’ option as Electrostatics. The Protonate 3D protocol was run using the default parameters in MOE, which include a physiological pH value of 7.0.

### 2.10 Unfolding Stability Change Prediction

The assessment of the changes in the unfolding stability (ΔΔG) was conducted via FoldX (Schymkowitz et al. 2005). The FoldX software was used to predict the change in protein stability (ΔΔG) upon mutation. The ΔΔG value was calculated using the formula ΔΔG = ΔG_var − ΔG_wt, where ΔG_var and ΔG_wt represent the calculated unfolding free energy of the variant and wild‐type structures, respectively. Thus, a negative ΔΔG value signifies a stabilizing mutation, while a positive ΔΔG value signifies a destabilizing mutation. The pipeline comprises three main steps. Firstly, ‘ReconstructSidechains’ function was applied to identify erroneous sidechains and reconstruct residues with missing atoms. Secondly, the reconstructed structures were optimized using the function ‘RepairPDB’. This function identifies those residues that have bad torsion angles, Van der Waals clashes, or total energy. Those residues will be mutated as well as their neighbors to the original amino acids themselves to explore different rotamer combinations to find new energy minima. Lastly, ‘BuildModel’ function was applied to the pre‐processed structure to compute for each variant a comparative model. Error was estimated by modeling each variant five to fifty times.

## 3 Results

### 3.1 Patient Phenotype

In a patient exhibiting recurrent bouts characterized by intestinal dysmotility, extrapyramidal movement disorder, and headaches from late infancy onwards, whole exome sequencing trio analysis revealed a de novo heterozygous nonsynonymous variant in SLC12A4 (c.3193G>A). This so‐called VUS in GUS (variant of unknown significance in a gene of unknown significance) encodes E1065K in KCC1.

### 3.2 Cell Biological Properties for KCC1 WT and E1065K Are Similar

**Figure 1:**
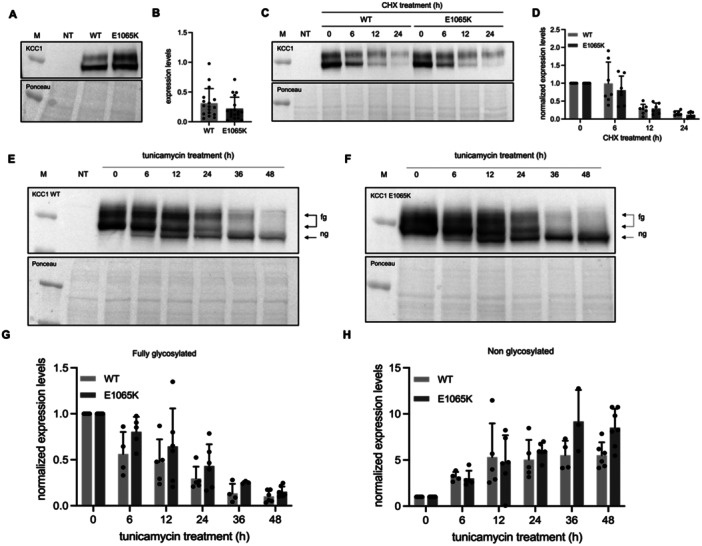
Cell biological properties of KCC1 WT/E1065K protein. Protein expression levels of KCC1 WT and E1065K (A) SDS‐PAGE result (Mw KCC1 = 120 kDa) and (B) total protein expression levels of KCC1 WT (*n* = 17) and E1065K (*n* = 17) transfected in HEK293T cells. Protein half‐life of KCC1 WT and E1065K (C) SDS‐PAGE result and (D) normalized expression levels of KCC1 WT (*n* = 7) or E1065K (*n* = 6–7) transfected in HEK293T cells and treated with cycloheximide (CHX, 200 µg/mL) for 0, 6, 12 or 24 h. N‐linked glycosylation of KCC1 WT and E1065K protein (E) and (F) SDS‐PAGE results and (G) and (H) normalized expression levels of KCC1 WT (*n* = 4–6) or E1065K (*n* = 5–7) transfected in HEK293T cells and treated with tunicamycin (5 µg/mL) for 0, 6, 12, 24, 36 or 48 h. NT = non transfected, M = marker, fg = fully glycosylated, ng = non glycosylated. Values are shown as mean ± SD.

Site‐directed mutagenesis was applied to produce the E1065K variant using the human WT KCC1 expression construct as a template. To explore full‐length protein expression of WT and E1065K KCC1, expression constructs were transfected into HEK293T cells. Subsequent Western blot analysis reveals the presence of two bands, potentially representing an immature and mature form of KCC1, Figure 1A. Quantification indicates no difference in expression levels between WT and E1065K, Figure 1B.

The two bands with different molecular weight, as in Figure 1A, might be the result of posttranslational modifications, or breakdown patterns. To investigate whether KCC1 WT and E1065K become glycosylated, transfected HEK293T cells were treated with tunicamycin (5 µg/mL), an N‐linked glycosylation inhibitor, for 0–48 h. We anticipated that by inhibiting glycosylation the heavier molecular weight band would disappear, and the lower weight form would become expressed stronger. Surprisingly, the two original bands, as in Figure 1A, both started to disappear upon tunicamycin treatment, and an additional lower molecular weight band appeared, Figure 1E,F. The two fully N‐glycosylated bands (fg) disappeared almost completely after 36h for both KCC1 WT and E1065K. The non‐glycosylated band (ng) started to appear within 6 h for KCC1 WT and E1065K. For KCC1 WT, the maximum amount of non‐glycosylated protein expression level was reached at approximately 12 h, Figure 1G. For KCC1 E1065K that was reached at approximately 36 h, Figure 1H. We conclude that the KCC1 protein is N‐linked glycosylated, and WT and E1065K variant have a similar rate of glycosylation.

To investigate the breakdown pattern of KCC1. HEK293T cells were transfected with KCC1 WT or E1065K DNA and treated with cycloheximide (200 µg/mL), an inhibitor of protein synthesis, for 0–24 h. Protein samples were analyzed with SDS‐PAGE western blotting, Figure 1C. Both KCC1 WT and E1065K, Figure 1D, showed a similar breakdown rate with a half‐life of approximately 8–10 h. Besides, in Figure 1C the heavier molecular band seems to be more stable than the lower molecular weight band. This difference in stability could suggest that the breakdown of the heavier molecular weight protein is slower. That could indicate that KCC1 undergoes additional posttranslational modifications, besides the N‐linked glycosylation, and therefore the more mature protein is less prone to breakdown, and/or the not fully matured protein still becomes mature.

### 3.3 KCC1 Localization under Endocytosis Inhibition

**Figure 2:**
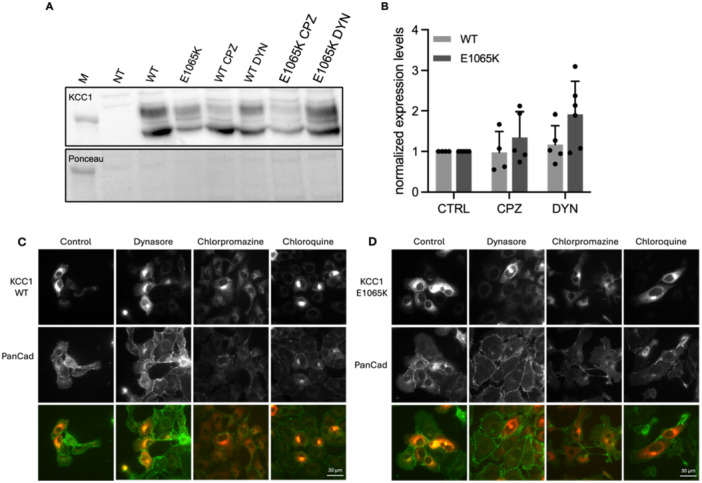
KCC1 WT/E1065K backward trafficking. (A) SDS‐PAGE results and (B) normalized expression levels KCC1 WT (*n* = 4–6) or E1065K (*n* = 5–6) transfected in HEK293T cells and without treatment (CTRL) or with either chlorpromazine (CPZ, 10 µM) or dynasore (DYN, 10 µM) treatment for 24 h. M = marker, NT = non transfected. Values are shown as mean ± SD. Immunofluorescence microscopy images of EPI7 cells transfected with (C) KCC1 WT and (D) E1065K, Flag (KCC1) in red, Pan‐Cadherin for membrane staining in green. Cells were treated with dynasore (10 µM), chlorpromazine (10 µM) or chloroquine (10 µM) for 24 h.

Since some changes in protein expression were apparent, although not statistically significant, it was decided to further investigate the backward trafficking of KCC1. KCC1 localization upon inhibition of clathrin‐coated endocytosis was determined with immunofluorescence microscopy, Figure 2C, D. To obtain a better view on all different steps of the clathrin‐mediated endocytosis pathway, chloroquine treatment was also included in the experiments. The immunofluorescence microscopy images showed that KCC1 is mainly expressed intracellularly with limited plasma membrane expression. At a few locations, some overlap between KCC1 protein (Flag in red) and plasma membrane (Pan‐Cad in green) seemed present. However, expression levels on the plasma membrane in general seemed limited and therefore make quantification of colocalization unreliable and therefore hard to establish. Upon dynasore treatment, the localization of KCC1 is still intracellular but seemed to be more clustered within the cell. This intracellular localization pattern became even stronger upon chlorpromazine and chloroquine treatment. In COS7 cells (see Figure S2), upon chloroquine treatment, localization in intracellular vesicle‐like structures became even more prominent compared to the treated EPI7 cells. This might indicate a build‐up of KCC1 in, for example, lysosomal structures. The above‐described patterns are seen for both KCC1 WT and E1065K, with no difference between the two. Combining the western blot and immunofluorescence microscopy data, this might indicate that KCC1 protein is degraded, at least partly, via the clathrin‐coated vesicle pathway.

### 3.4 Modeling of KCC1 Protein Reveals Changes in Interactions With E1065K

With respect to experimentally solved structures of KCC1 in the Protein Data Bank (last accessed Jan. 2023), three groups (Liu et al. 2019; Chi et al. 2021; Zhao et al. 2022) released Cryo‐EM structures of KCC1. Considering the location of the variant on the C‐terminal domain (CTD) as well as the general structure quality, two PDB structures (PDB ID: 7AIP, 7AIR) were used as templates for the following modeling processes.

Both structures were captured as dimers (chain A and chain B) in an inward‐facing (IF) conformation with ATP co‐crystallized in the CTD. The major differences we observed between these two structures were the interactions formed between E1065 and N1026, as well as an additional fraction of flexible chain spanning between helices a10 and a8 present only in the chain A of 7AIR. In 7AIP, chain B has a higher quality than chain A regarding the geometric issues observed across the polymeric chains and their fit to the map. Hence, 7AIR chain A and 7AIP chain B are selected as templates for the following modeling.

**Figure 3:**
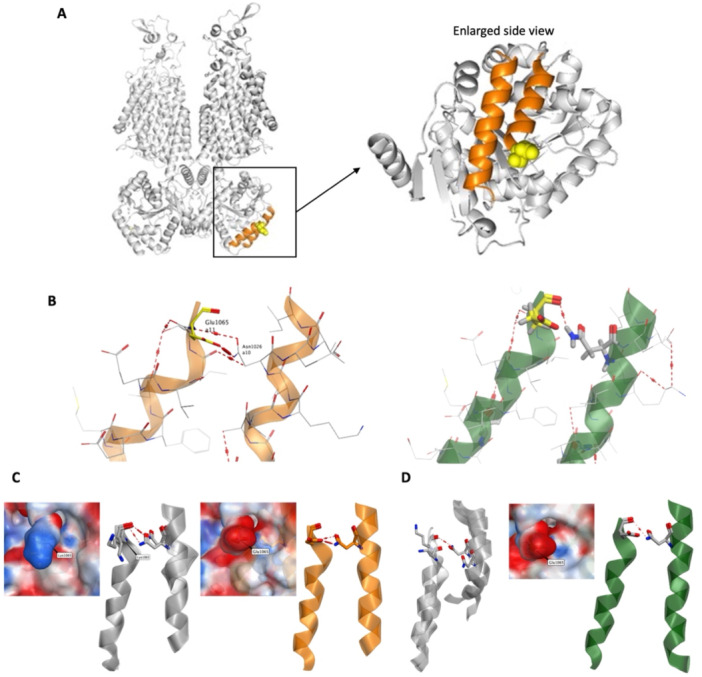
Interactions observed in the experimentally solved KCC1 structures. (A) KCC1 structure with focus on the area around E1065 with alpha helix 10 and 11 highlighted (B) In orange the PDB ID 7AIP. In green, the PDB ID 7AIR structure. (C) The electrostatic map around the variant E1065K, as well as the original residue E1065. The electronic donor is mapped with the blue surface and the acceptor with red. While the experimentally solved structure (7AIP) is colored in orange, the correspondent models from both the standard and the enhanced script are colored in gray. The model from the enhanced script demonstrated a rotation of the K1065 sidechain toward the lower part of the helix. (D) The local electrostatic map around the E1065 from the structure with PDB ID 7AIR. The electronic donor is mapped with the blue surface and the acceptor with red. The PDB structure is colored in green, whereas the correspondent models are in gray.

The interaction pattern of the wild‐type residue E1065 is presented differently in these two structures. Two interactions were observed between the 7AIP chain B (7AIP_B, hereafter referred to as 7AIP), while in the 7AIR chain A (7AIR_A, hereafter referred to as 7AIR) only one was observed (Figure 3A). The interactions between E1065 and N1026 are the main interactions observed between helixes a10 and a11.

With the chosen structural templates, 7AIP and 7AIR, the conformer of the variant E1065K was modeled. When analyzing the model with 7AIP as the template, the same observations were made from both the standard and the enhanced protocol, where the model optimization and refinement were leveraged. In the experimentally solved structure (7AIP), a sidechain and a backbone interaction were observed between E1065 and N1026. In the correspondent model of the variant, the sidechain interaction was rejected, whereas the backbone interaction remained. The K1065 in the variant model demonstrated a rotation away from the helix a10 (Figure 3B). This observation can be supported by the following two factors: firstly, the sidechain of lysine possesses the opposite charge from the one of glutamate, which is not favored towards the secondary nitrogen of the amide group from the original interaction partner asparagine (N1026). Secondly, the electrostatic map of the variant model suggests that the local environment of the helix a11 comprises an enriched electron density beneath the K1065, which is in favor of the positive‐charged lysine. The second factor is well‐represented in the enhanced model with 7AIP as the template. The model with 7AIR as the template provided some interesting perspectives. In 7AIR, only a weak backbone interaction can be observed between E1065 and N1026. As for the correspondent variant models, different observations were made from the standard procedure and the enhanced one. The standard procedure discarded the backbone interaction by pushing the sidechain of N1026 away from a plausible distance for potential H‐bond forming. Concerning the enhanced optimization and refinement, the model from the enhanced procedure withheld the backbone interaction between K1065 and N1026 by flipping the amide group of the asparagine. Moreover, a bent conformation of helix 10 appeared in both outputs of the standard and enhanced protocols with 7AIR as the template (Figure 3C). This can be induced by an increased flexibility of the additional fraction of linker between a10 and a8 present in 7AIR.

The changes in the unfolding stability (ΔΔG) between the WT structure and the variant were accessed with FoldX. An error margin is suggested to be considered in the interpretation of the analysis of the *in silico* prediction of the FoldX. Van Durme et al (Van Durme et al. 2011) recommended 0.5 kcal/mol in their work of integrating FoldX for YASARA, whereas Buß et al. (Buß et al. 2018) regarded a stabilizing variant with a ΔΔG value between −0.75 and −5 kcal/mol, a destabilizing one with a value greater than 1 kcal/mol.

The aforementioned two templates were utilized in the prediction as they offer different perspectives of possible interaction patterns between the residues of interest. Both procedures resulted in negative ΔΔG values slightly over the suggested error margin with five run times. When increase the run times to fifty, no significant changes of the result were observed (Table 1).

**Table 1:** *In Silico* Unfold Stability Changes Prediction of the FoldX.

Template	Runs	ΔΔG [kcal/mol]	Runs	ΔΔG [kcal/mol]
7AIP	5	−0.89	50	−0.86
7AIR	5	−0.96	50	−1.06

### 3.5 Reduction of KCC1 E1065K Function under Hypotonic Condition

To assess whether the KCC1 variant impacts ion‐transport activity, we employed an ammonium assay designed for potassium‐chloride co‐transporter (KCC) activity measurement (Medina and Pisella 2020). This assay monitors intracellular pH (pH_i_) changes following NH_4_^+^ application to cells, as NH_4_^+^ ions serve as surrogates for K^+^ ions normally transported by KCC1. Consequently, the kinetics of pH_i_ changes provide an indirect measure of KCC1 ion‐transport activity.

KCC1 exists in seven isoforms generated by alternative splicing (KCC1‐1 to KCC1‐7) (Kok and Brodsky 2024). For functional testing, HEK293T cells were transfected with expression vectors encoding either the KCC1‐1 isoform, which is the largest and most ubiquitously expressed canonical variant, or the KCC1‐2 isoform, previously used for biochemical and structural investigations, together with their respective E1065K variant. The same cells were co‐transfected with a ratiometric, pH‐sensitive fluorescent probe to monitor NH₄⁺‐induced changes in pH_i_.

**Figure 4:**
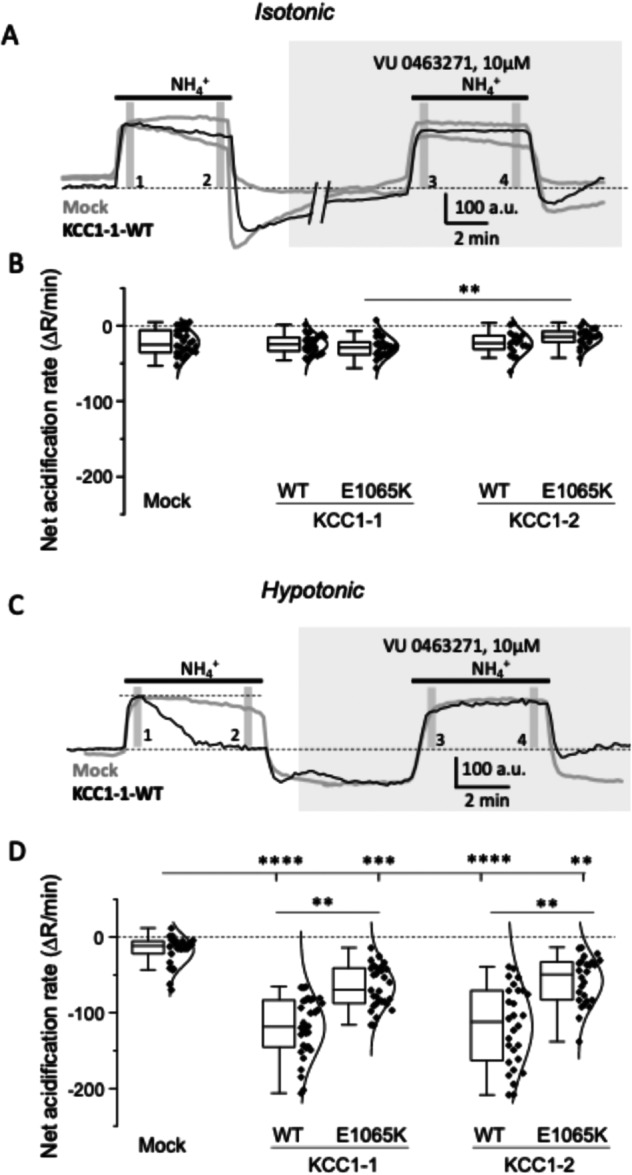
Functional NH₄⁺ flux experiments on KCC1‐1 and KCC1‐2 isoforms (wild type, WT) and their E1065K variant transiently expressed in HEK293T cells. (A) Representative longitudinal recordings under isotonic conditions showing the ratio of fluorescence emitted by pHluorin and mCherry (Fₚ_H_/Fₘ_Ch_) in two mock‐transfected cells from different experiments (to illustrate variability) and a typical trace from a KCC1‐1_WT–expressing cell. Horizontal bars indicate the duration of application of recording solutions containing 10 mM NH₄⁺, and 10 mM NH₄⁺ plus 10 μM VU0463271. Vertical bars 1–4 mark the time points used to measure Fₚ_H_/Fₘ_Ch_ values for quantifying the rate of pH‐dependent fluorescence decay. (B) Tukey box plot of net VU0463271‐dependent acidification rates in isotonic conditions for the indicated constructs. Rates were obtained by subtracting the difference between points 3 and 4 (panel A) from the difference between points 1 and 2. The Kruskal–Wallis test revealed a significant difference across the dataset (*p* = 0.016, χ² = 12). Post hoc Dunn's test indicated a significant difference between KCC1‐1_E1065K and KCC1‐2_E1065K (*p* = 0.008; *N* = 4, *n* = 29 and 21, respectively). (C) Representative longitudinal recordings under hypotonic conditions. Same legend as in panel A applies. Note the rapid decay of Fₚ_H_/Fₘ_Ch_ in KCC1‐1_WT–expressing cell compared with mock‐transfected cell and the inhibition of this decay in the presence of VU0463271. (D) Tukey box plot of net VU0463271‐dependent acidification rates in hypotonic conditions. Values were calculated as described for panel B. The Kruskal–Wallis test revealed a significant difference across the dataset (*p* < 0.0001, χ² = 87). Post hoc Dunn's test indicated significant pairwise differences as shown on the plot. *N* = 4; *n* = 30 (mock), 32 (KCC1‐1_WT), 31 (KCC1‐1_E1065K), 28 (KCC1‐2_WT), 26 (KCC1‐2_E1065K). (*p *< 0.01 for **p < 0.001 for ***p < 0.0001 for ****). The Y‐axes in panes B and D are identical to allow direct comparison of the values under isotonic and hypotonic conditions.

Upon exposure to 10 mM NH₄⁺ under isotonic conditions, mock‐transfected cells exhibited a rapid cytoplasmic alkalinization followed by either a gradual acidification or stabilization during NH₄⁺ application, depending on individual cells and experimental batches (Figure 4A). Cells expressing any of the KCC1 constructs displayed similar response profiles. Application of 10 μM VU0463271, a selective inhibitor of K⁺–Cl⁻ cotransporters (Delpire et al. 2012) that binds with high affinity to KCC1 (Zhao et al. 2022), significantly reduced the acidification magnitude in both mock‐transfected cells and those expressing KCC1 constructs (Figure 4A, Supplementary Figure S3A). The magnitude of VU0463271‐induced inhibition did not differ significantly between mock‐transfected cells and those expressing WT or E1065K KCC1 constructs (Figure 4B). We therefore concluded that, under isotonic conditions, overexpressed KCC1 isoforms or their variants do not produce detectable ion flux, consistent with the well‐established inactivity of KCC1 under isotonic conditions (Mercado et al. 2000).

Under hypotonic conditions, NH₄⁺ exposure in mock‐transfected cells produced a similar rapid alkalinization followed by slight or no acidification, mirroring the isotonic response (Figure 4C vs Figure 4A). In contrast, cells expressing WT KCC1 exhibited a pronounced acidification phase (Figure 4C), which was effectively abolished by 10 μM VU0463271 (Figure 4C, Supplementary Figure S3B). The mean values of the net VU0463271‐sensitive acidification component under hypotonic conditions were more than six‐fold greater for both KCC1‐1 WT and KCC1‐2 WT compared with mock‐transfected cells (*p* < 0.0001 for both isoform 1 and 2, Figure 4D). Overexpression of the E1065K variant (KCC1‐1_E1065K and KCC1‐2_E1065K) produced intermediate responses, but significantly smaller (isoform 1 *p* < 0.001 and isoform 2 *p* < 0.01) than those observed for WT constructs but higher than those of mock‐transfected cells. In conclusion, the E1065K variant impairs KCC1 function as measured under hypotonic conditions.

## 4 Discussion

In this study, the (sub)cellular expression, localization and function of KCC1 WT and E1065K were investigated. We uncovered that the function of the KCC1 E1065K patient variant, compared to WT, is decreased under hypotonic conditions due to decreased activation. A change in activation of the cotransporter can have a large impact on the integrity of cells and, therefore, may be pathogenic. Furthermore, the cell biological properties of KCC1 are not abundantly studied and the knowledge on (sub)cellular handling of this protein is limited. The data in this study contribute to fill this gap, by uncovering the half‐life, glycosylation patterns and trafficking pathway.

For the analysis of KCC1 ion‐transport and biochemical properties, we used the HEK‐293 cell line, which is widely employed for studying KCC transporters as well as many other ion transporters and channels. A characteristic feature of HEK‐293 cells is their expression of a broad spectrum of endogenous ion channels and transporters that should be taken in consideration (Zhang et al. 2022). To minimize competition with KCC1 for K⁺ flux, we routinely applied ouabain, amiloride, and bumetanide to inhibit the endogenous Na⁺/K⁺‐ATPase, Na⁺/H⁺ exchanger, and NKCC1, respectively. Despite the presence of these inhibitors, a small endogenous component mediating NH₄⁺ extrusion was detected in mock‐transfected cells under both isotonic and hypotonic conditions. Similar background activity has been reported in HEK‐293 cells under isotonic conditions using either Rb⁺ flux assays (Hartmann et al. 2010) or NH₄⁺‐based assays (Hershfinkel et al. 2009). In our experiments, this endogenous flux was sensitive to VU0463271. The origin of this background VU0463271‐sensitive flux remains unclear and requires further investigation. It may arise from one or more endogenous KCC isoforms (KCC1, KCC3, or KCC4), from other cation transporters resistant to bumetanide and amiloride, or from one of the many potassium channels expressed endogenously in HEK‐293 cells (Zhang et al. 2022). Although VU0463271 is considered a selective KCC inhibitor (Delpire et al. 2012), the breadth of its activity has not been fully characterized, particularly with respect to ion channel inhibition. Because the magnitude of VU0463271‐dependent pH responses under isotonic conditions was indistinguishable between mock‐transfected cells and those expressing either wild‐type or E1065K KCC1, and given the > 6‐fold increase in VU0463271‐sensitive KCC1 activity under hypotonic conditions, we conclude that overexpressed KCC1, whether wild‐type or mutant, is inactive under isotonic conditions and becomes activated only in response to hypotonic stress.

KCC1 expression and function have been investigated in the context of human diseases like e.g., sickle cell disease (Pan et al. 2011) and cancer (Zhang et al. 2009), but thus far, KCC1 was never linked to human disease (Garneau et al. 2019). Most studies that are investigating the connection between KCC1 and disease are performed in cell models, like HEK293 or human erythroid K652 cells, or animal models, mostly mouse knock‐out (Shmukler et al. 2019, 2020). We were able to find one study that investigates a point mutation that was predicted to affect phosphorylation in a humanized mouse model and HEK293 cells (Brown et al. 2015). The study described in this paper is, as far as we know, the only study that shows the link between a rare variant found in a patient and an alteration in KCC1 function.

With respect to other members of the KCC family, pathogenic variants have been identified previously. Notably, during the last decade, a number of pathogenic variants were described in KCC2 (Fukuda and Watanabe 2019; Järvelä et al. 2024), like the KCC2 R231H missense variant that leads to epilepsy. Interestingly, this loss‐of‐function variant shows a similar functional decrease as our KCC1 variant with NH_4_^+^ flux assay (Järvelä et al. 2024). Also, different KCC3 de novo variants were discovered that resulted in Charcot‐Marie‐Tooth disease, a progressive sensorimotor neuropathy (Park et al. 2020).

The modeling of KCC1 in this study showed that the variant, E1065K, changes the interaction observed in the experimentally solved structures between helices a10 and a11. The original interactions formed in the WT structure are the only interactions that rigidifies a10 to a11. The E1065K variant disrupts this interaction, which is locally destabilizing. The replacement of a negatively charged glutamate with a positively charged lysine in an enriched electron density environment likely reduces local electrostatic repulsion. Concurrently, the loss of this constraining bond increases conformational flexibility in the C‐terminal domain. The net result is a ΔΔG value of around −1 kcal/mol, is best interpreted as a neutral overall stability change. This aligns with the functional data, which shows a properly trafficked protein with specific regulatory deficits, rather than global destabilization.

Since KCC1 is not studied extensively, it is interesting to look at other CCC members. The complete conservation of residues E1065 and N1026 in KCC2 and KCC3 provides a strong rationale to investigate whether the destabilizing mechanism we identified extends to other KCC paralogs, directing an important avenue for future analysis. Furthermore, a recent structure of activated NKCC1 reveals a regulatory mechanism involving phosphorylation‐dependent binding of the N‐terminal domain to the CTD (Zhao et al. 2025). The KCC1 E1065 residue maps to a location on the CTD (K1192 in NKCC1) that is distinct from this primary NTD‐binding interface. This suggests the E1065K variant may not directly affect this particular mechanism. However, its impact on KCC1 function underscores the CTD's complex role in regulation, which likely involves additional, yet‐to‐be‐defined interaction surfaces.

Since KCC1 cell biological properties are scarcely investigated they are therefore not widely described in the literature. The results in this study regarding KCC1 protein cell‐biology aid in understanding the properties of the cotransporter. Our findings on KCC1 biochemistry and cell biology display much overlap with those of other KCC members. KCCs have a distinctive long extracellular loop between transmembrane domains 5 and 6 with glycosylation sites. Although the loop is poorly conserved, these glycosylation sites are conserved between KCCs and are not present in other CCCs (Mount et al. 1999). In KCC4 N‐linked glycosylation sites are important for maturation and trafficking of the protein (Weng et al. 2013). In KCC3 a similar glycosylation pattern is seen as in this study, for both a WT and a variant protein. KCC3 western blot results also show that upon tunicamycin treatment, the two different heavier molecular weight bands disappear and a lower weight band appears (Ding et al. 2013). Furthermore, our study showed a half‐life between 8 and 10 h for KCC1 protein. KCC4 has a comparable half‐life as the results presented in this paper; the lower molecular weight, less mature, band has a half‐life also in the range of hours and the mature, higher molecular weight, more mature, band is somewhat decreased in 12 h (Weng et al. 2013). For KCC2 there is no consensus on the protein half‐life, it ranges between a few minutes to even hours or days. Obviously, these results come from different research groups with different methods for quantifying the rate of degradation (Rivera et al. 2004; Medina et al. 2014; Lee et al. 2011; Puskarjov et al. 2012). KCC2 is internalized via clathrin coated endocytosis and degraded via the lysosome (Zhao et al. 2008; Lee et al. 2010). However, KCC4 is degraded via proteasomal degradation, since inhibition with proteasomal inhibitor MG132 only showed a decrease in the amount of KCC4 protein, and lysosomal and protease inhibitors did not (Weng et al. 2013).

The KCC1 C‐terminus contains partially conserved phosphorylation sites (Rinehart et al. 2009; Zimanyi et al. 2020) and an ATP/ADP binding site (Chi et al. 2021). Phosphorylation of KCC1, through SPAK/OSR1 protein kinases, inhibits ion transport via the cotransporter (Rinehart et al. 2009; de los Heros et al. 2014). Furthermore, it would be worthwhile to explore the effect of the variant in red blood cells, since this is one of the cell types in which KCC1 is most described. Eventually, the step towards a model that represents the whole physiology should be considered. Since KCC1 is abundantly expressed in many different tissues and the symptoms of the described patient are diverse and probably effecting different parts of the body. The results of this study at least warrant further research in different models to further increase comprehension of this variant.

In this study, we showed the first human potential pathogenic variant in the KCC1 protein. Although this study was performed in ectopic overexpression systems, and more research is needed to make an irrefutable conclusion, this variant is pathogenic and disease causing. The reduction of activation of KCC1 E1065K under stressful conditions for the cell is a promising start of understanding how this variant is linked to pathology in humans.

## Author Contributions

Conceptualization: Marcel A. G. van der Heyden, Peter M. van Hasselt, Meye Bloothooft; Formal analysis: Meye Bloothooft, Jiahui Huang, Mira Hamze, Teun P. de Boer, Christophe Porcher, Marcel A. G. van der Heyden; Investigation: Meye Bloothooft, Jiahui Huang, Mira Hamze, Marien J. C. Houtman, Christophe Porcher; Methodology: Meye Bloothooft, Jiahui Huang, Mira Hamze, Teun P. de Boer, Gerhard F. Ecker, Christophe Porcher, Igor Medina, Marcel A. G. van der Heyden; Project administration: Meye Bloothooft, Marcel A. G. van der Heyden; Resources: Meye Bloothooft, Jiahui Huang, Mira Hamze, Marien J. C. Houtman; Supervision: Teun P. de Boer, Gerhard F. Ecker, Christophe Porcher, Igor Medina, Marcel A. G. van der Heyden; Validation: Meye Bloothooft, Jiahui Huang, Mira Hamze, Marien J. C. Houtman, Teun P. de Boer, Peter M. van Hasselt, Gerhard F. Ecker, Christophe Porcher, Igor Medina, Marcel A. G. van der Heyden; Visualization: Meye Bloothooft, Jiahui Huang, Mira Hamze; Writing—original draft: Meye Bloothooft, Jiahui Huang, Mira Hamze, Marcel A. G. van der Heyden; Writing—review & editing: Meye Bloothooft, Jiahui Huang, Mira Hamze, Marien J. C. Houtman, Teun P. de Boer, Peter M. van Hasselt, Gerhard F. Ecker, Christophe Porcher, Igor Medina, Marcel A. G. van der Heyden.

## Funding

This research did not receive any specific grant from funding agencies in the public, commercial, or not‐for‐profit sectors.

## Conflicts of Interest

The authors declare no conflicts of interest.

## Declarations

This work does not contain Artificial Intelligence Generated Content (AIGC).

## Supporting information

supmat.

## Data Availability

All data is available from the corresponding author upon request.
