# Supplementary material for: Exploring the Cell Biological and Functional Effects of the First Disease Associated KCC1 Genetic Variant
Source: J Cell Physiol. 2025 Dec 19;240(12):e70124. doi: 10.1002/jcp.70124 (PMC12715662; doi:10.1002/jcp.70124)
Supplement: Supplementary file 1 — supmat. [file JCP-240-0-s001.docx]

**Supplemental data**

**Supplementary Figure S1. KCC1 protein expression in different cell lines.**

**
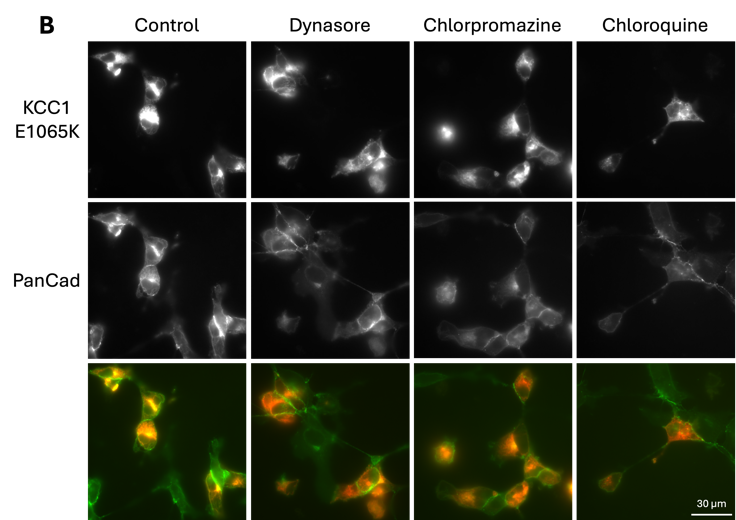
**


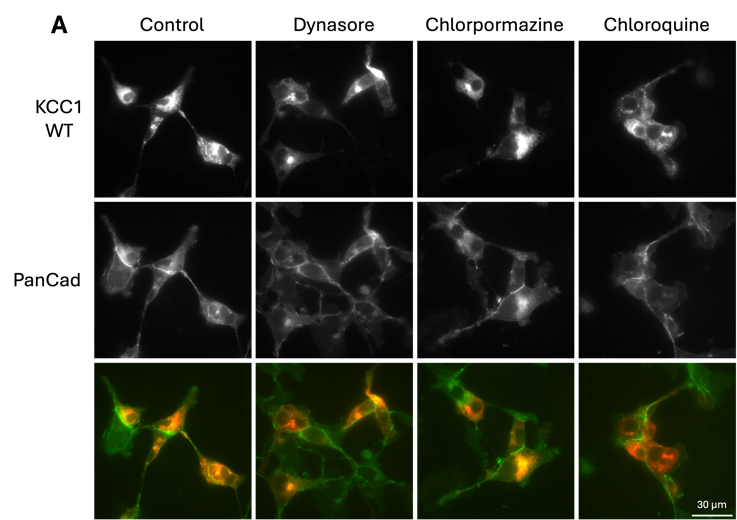


**Supplementary Figure S2.** Immunofluorescence microscopy images of COS7 cells transfected with KCC1 A) WT and B) E1065K, Flag (KCC1) in red, Pan-Cadherin for membrane staining in green. Cells were treated with dynasore (10 µM), chlorpromazine (10 µM) or chloroquine (10 µM) for 24 hours.

**Supplementary Figure S3. Effects of the KCC inhibitor VU0463271 on NH₄⁺-induced fluorescence decay in HEK293T cells expressing KCC1-1 and KCC1-2 isoforms (wild type, WT) and their E1065K variant.**

(A) Tukey box plot of acidification rates during exposure to 10 mM NH₄⁺ under isotonic conditions for the indicated constructs. Quantification is based on traces similar to those shown in Figure 4A. Rates were calculated by subtracting the difference between points 1 and 2 (control) and points 3 and 4 (VU0463271-treated), respectively, see Figure 4. The Kruskal–Wallis test revealed a significant difference across the dataset (P < 0.0001, χ² = 160). Post hoc Dunn’s test indicated significant differences between control and VU0463271-treated values in all conditions (P < 0.0001 for ****; P = 0.006 for **; P = 0.02 for *). N = 4; n = 32 (mock), 29 (KCC1-1_WT), 29 (KCC1-1_E1065K), 20 (KCC1-2_WT), 21 (KCC1-2_E1065K). (B) Tukey box plot of acidification rates during exposure to 10 mM NH₄⁺ under hypotonic conditions. Quantification is based on traces similar to those in Figure 4C. Rates were calculated as in panel A. The Kruskal–Wallis test revealed a significant difference across the dataset (P < 0.0001, χ² = 221). Post hoc Dunn’s test indicated significant differences between control and VU0463271-treated values in the conditions indicated with asterisks (P=0.45 for ns; P < 0.0001 for ****). N = 4; n = 32 (mock), 30 (KCC1-1_WT), 31 (KCC1-1_E1065K), 28 (KCC1-2_WT), 26 (KCC1-2_E1065K). The Y-axes in panes A and B are identical to allow direct comparison of the values under isotonic and hypotonic conditions.
